# A Fatal Case of Hæmophilia

**Published:** 1900-05

**Authors:** Thomas Fillebrown


### A FATAL CASE OF HÆMOPHILIA.[1]

BY THOMAS FILLEBROWN, M.D., D.M.D.

A PATIENT, male, aged twenty-five years, was suffering from an alveolar abscess on the distal root of the left inferior first molar, which was discharging through a fistula on the side of the face near the lower border of the under jaw. The abscess was of three years' standing. The constant discharge from the abscess had become so exceedingly offensive that the patient felt he must have it relieved. He was brought to me by Dr. L. G. Forrest for operation, April 26, 1899.

Some months previous Dr. Forrest had consulted me about the case, and had given me quite a full description of the trouble. I then advised that when the patient decided to have the tooth removed, he should take a course of astringent tonic for some two weeks just previous to coming for the operation.

I based this advice upon the fact that I had in several cases pursued this plan with my own patients, and the best of results had followed; and had the state of the blood in this case been the only condition unfavorable, it evidently would have proved sufficient, for the blood proved to be readily coagulable, forming a clot firm and disposed to be adherent; and had the arteries had any contractile power, I am sure success instead of defeat would have been the result.

On Wednesday, April 26, I extracted the roots of the first and also of the second molar, all of which were decayed to the gum and quite loose. The roots were removed without difficulty. The bleeding was somewhat profuse, but not excessive, and soon ceased. After a time he went out a short distance and took a light lunch. He returned to my office, and in about an hour and a half after the operation the blood commenced to flow again.

I plugged the sockets with cotton and Monsel's persulphate of iron, but I could not control the bleeding. I then took a plaster impression of the under jaw and made a hard rubber jacket plate, which, when applied with a layer of gauze under it and held in place by a firm head bandage, controlled the bleeding apparently

[1] Read before the American Academy of Dental Science, December 9, 1899.

for six hours or more, when it had to be readjusted, as the blood had worked its way out under the compress.

He then went to the Elliott Hospital, in order that he might have careful nursing and timely attention.

The second adjustment of the splint held the blood in check for about six hours more, when the blood flowed freely again.

At noon of Thursday, the 27th, as the upper teeth had become quite sore, I concluded to try the Harvard dental splint, and with the assistance of Mr. Curry, one of the students of the Harvard Dental School, made a jacket and applied the splint at five o'clock P.M. This seemed to promise success, as it held the blood in check for twenty-two hours, when this also failed.

I then packed with gauze and cotton, holding it down by the upper teeth. I renewed these packings every five or six hours, as that was as long as any one application would serve; and the large amount of clot that came from the mouth and throat when the packings were removed showed plainly that the flow of blood had only been retarded and not stopped.

On Sunday, April 30, I decided to try the actual cautery, and at about three o'clock, with the assistance and advice of Dr. F. W. Rice, I thoroughly cauterized the bleeding surface, which was confined to the edge of the gum opposite the mesial root of the first molar on the lingual side. A firm clot covered the other parts of the wound and completely stopped the blood. The cautery, supplemented by nitrate of silver, completely checked the flow of blood, and we thought the victory won. In about an hour the blood-pressure removed the eschar, and the blood again flowed as freely as ever.

I resorted again to the compress, but had more difficulty in controlling the hemorrhage. At eleven o'clock P.M. I called Dr. Brewster, who advised with me, making valuable suggestions and rendering assistance, which kept the trouble fairly well controlled through the night. Dr. C. A. Porter was also present, and at his suggestion and advice I gave the patient a full course of chloride of calcium. The effect was not so favorable as we had hoped. Previous to this I had administered ergot in full doses for nearly twenty-four hours, with negative results.

On Monday, May 1, at noon, acupressure was resorted to. I secured the services of Messrs. Wentworth and McHale, dental students, who kindly volunteered for the service, and faithfully and skilfully maintained pressure during the afternoon and night, but could not succeed in wholly arresting the flow.

On Tuesday, May 2, I was called at two o'clock A.M., as the patient seemed sinking, but it proved to be fainting; and as he seemed too weak to bear more manipulation, the packing and pressure were not resumed.

The only hope now lay in the natural cessation of the flow of blood, as had occurred before when the loss of blood was excessive, and as frequently occurs in similar cases. But it was of no avail, and at 1.30 P.M., Tuesday, May 2, the patient passed away, a victim to the loss of blood. Blood continued to flow from the wound after the pulse at the heart had stopped and the breathing had nearly ceased.

On Monday evening the infusion of a saline solution was considered, and Dr. Brewster was present prepared to perform it. Dr. Porter was also present. Upon consideration, it was deemed unadvisable, as it would add another wound, and the salt would make the blood less coagulable and offer no compensating stimulus to the nervous system which would serve to contract the vessels. The lack of contractility of the arteries seemed to be the main trouble, as the blood formed a very firm clot.

The undertaker gave me the following statement: "I found the arteries in a very abnormal condition. Neither myself nor my assistant could find any trace of the femoral artery or its sheath, and after repeated attempts, gave it up and sought for the left brachial artery, which I found.

"The division of the artery occurred several inches above the normal point. There was hardly a semblance of a sheath, and an almost total absence of the middle coat, which made the artery hardly distinguishable from the vein.

"The walls of the artery were so tender that the pressure of a finger was sufficient to tear it open, whereas in a normal case this is impossible, it often requiring the aid of a scalpel to extend the opening so as to admit the embalming syringe.

"The embalming was done less than six hours after death."

Following is the history of the patient: Paternal grandmother suffered from excessive nose-bleed. Had nares plugged to stop it more than once. Patient's father did not inherit the conditions. Patient's mother inclined to bleed freely.

At seven years of age he suffered from a slight injury to his left knee, which caused a scratch two or three inches long, but not through the skin. The part swelled enormously until the skin along the line of the scratch burst open. Bleeding followed, but not profuse, which soon reduced the swelling. It was more than a week before the bleeding ceased.

When about twenty-three years old he had a tooth extracted, and for nine days the blood flowed constantly, but not enough to keep him from his work. The same year his lip was injured quite severely. It swelled excessively, and bled for three weeks at times before it could be stayed. Ice with a spring clip was the last thing applied. He bled almost to collapse.

When twenty-four the patient had a portion of a tooth hanging by a little gum tissue. It was removed by the pressure of a finger. The bleeding continued long and was stopped with difficulty.

The more noticeable features of this case are the peculiar conditions of the arteries and the progressiveness of the disease. When a baby there was but little trouble, but at seven years of age the disease had become serious, and gradually increased until at twenty-five it proved irremediable.

